# Precise Drilling of Holes in Alumina Ceramic (Al_2_O_3_) by Rotary Ultrasonic Drilling and its Parameter Optimization using MOGA-II

**DOI:** 10.3390/ma13051059

**Published:** 2020-02-27

**Authors:** Hisham Alkhalefah

**Affiliations:** Raytheon Chair for Systems Engineering (RCSE), Advanced Manufacturing Institute, King Saud University, Riyadh 11421, Saudi Arabia; halkhalefah@ksu.edu.sa

**Keywords:** ceramics, rotary ultrasonic drilling (RUD), central composite design (CCD), multi-objective genetic algorithm (MOGA-II), aluminum oxide

## Abstract

Alumina is an advanced ceramic with applications in dental and medical sciences. Since ceramics are hard and brittle, their conventional machining is expensive, arduous, and time-consuming. As rotary ultrasonic machining is among the most adequate and proficient processing techniques for brittle materials like ceramics. Therefore, in this study, rotary ultrasonic drilling (RUD) has been utilized to drill holes on alumina ceramic (Al_2_O_3_). This study investigates the effect of key RUD process variables, namely vibration frequency, vibration amplitude, spindle speed, and feed rate on the dimensional accuracy of the drilled holes. A four-variable three-level central composite design (thirty experiments on three sample plates) is utilized to examine the comparative significance of different RUD process variables. The multi-objective genetic algorithm is employed to determine the optimal parametric conditions. The findings revealed that material removal rates depend on feed rate, while the cylindricity of the holes is mostly controlled by the speed and feed rate of the spindles. The optimal parametric combination attained for drilling quality holes is speed = 4000 rpm, feed rate = 1.5 (mm/min), amplitude = 20 (µm), and frequency = 23 (kHz). The validation tests were also conducted to confirm the quality of drilled holes at the optimized process parameters.

## 1. Introduction

Ceramics can be categorized as inorganic, crystalline, nonmetallic materials prepared from compounds of metal and nonmetal [1,2]. These materials, by virtue of their remarkable properties, command numerous applications in the automotive, aerospace, energy, electronics, and medical industries. They are impressive because they exhibit chemical inertness, preferable electrical, and magnetic properties, enhanced corrosion resistance, superior strength, exceptional wear resistance at higher temperatures, etc. [3,4]. Refer to remarkable electrical (insulating properties, electrical conductivity, dielectric strength, piezoelectric properties, etc.) and magnetic properties (permeability, retentivity or magnetic hysteresis, coercive force, and reluctance), the ceramics are used extensively in electronic/optical devices as well as being applied as high-quality films to base substrates [5]. Simultaneously, they are also considered challenging materials as a result of their brittle nature, creep resistance, and higher hardness [6,7,8,9]. They can be classified as hard to machine materials by conventional processing processes, namely lathe, milling, etc. Certainly, the poor machinability and inefficient machining performance limit their further industrial applications. It emphasizes the significance of competent and adequate machining processes relevant to advanced ceramics. 

**Figure 1:**
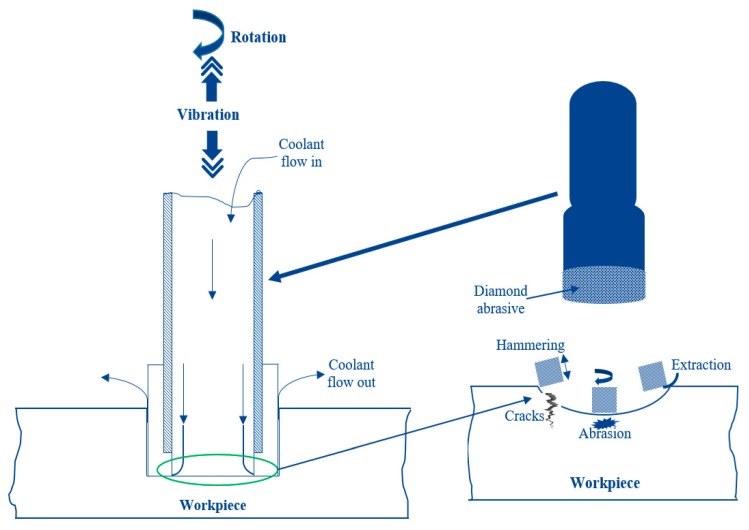
Rotary ultrasonic machining (RUM) working principle for material removal.

As one of the competent fabricating approaches for leading ceramics, rotary ultrasonic machining (RUM) has drawn profuse interest from the manufacturing industries [10]. It can be described as a hybrid processing technique, which integrates the conventional grinding and ultrasonic machining [11]. Laser machining is also popular with machine ceramics, however, there are numerous issues associated with it such as a larger taper angle, agglomeration of slag, and generation of the recast layer to name a few [12]. Among the current nontraditional material removal techniques, the RUM has been the most economical, eco-friendly, extensible, and applicable for intricate shapes due to its 5-axis machining proficiency. As shown in Figure 1, a rotating core drill coated with metal bonded diamond abrasives is ultrasonically vibrated and provided against the machining specimen with a steady feed rate. The coolant is also passed through the drill core to wash away the debris, as well as preventing the jamming and heating of the drill. The scheme of the material removal during RUM can be categorized into three main stages, that is (i) the hammering stroke of the tool due to ultrasonic vibrations causing indentation and crushing, (ii) abrasion, because of the rotational movement of the cutting tool, and (iii) withdrawal, the integrated action of ultrasonic vibrations and rotation of the tool results into this [13]. Note that the ultrasonic tools such as ultrasonic drills are designed so that they can resist the additionally generated oscillating motional kinematics in the axial direction, which results due to the employment of supersonic. As a result, these rotary ultrasonic drilling (RUD) tools possess unique technical properties of bond hardness, enduring material of the bonding matrix, grain quality, and diamond coat density.

The RUM can be classified as one of the most pertinent techniques to process difficult brittle materials, essentially ceramics and composites [14]. For example, Ishikawa et al. [15] employed a drilling approach that unified ultrasonic vibration of the diamond core drill and low-frequency vibrations of the specimen. They observed this approach was highly productive in drilling hard and brittle materials. Similarly, Pei et al. [16] performed machining of ceramics using RUM and recognized admirable surface quality in contrast to traditional machining. Moreover, Lv et al. [17] utilized the RUM technique on machining glass BK7 and noticed a convincing reduction in cutting force and improved machining performance. The RUM exhibits many benefits, such as higher production rate (ten times faster), superior hole accuracy, uncomplicated drilling of deep and small holes, excellent surface finish, etc., in comparison to conventional ultrasonic machining (USM) [18]. There are issues with ultrasonic machining also, such as due to stress concentration at the periphery of the hole exit a fracture at the edges occurs [19]. Wang et al. [20] employed a unique blended step-taper diamond core drill for RUM of C/ SiC to enhance the hole exit accuracy. The results indicated that the compound drill could be useful to minimize the tearing size by thirty percent. Due to the reprocessing effect of the compound drill the thrust force progressively reduces at the hole exit, and thus improves the quality.

However, the many benefits of RUM can only be actualized through the proper control of its various process parameters. Since there are considerable factors in RUM, therefore, they need to be adjusted to obtain their greatest relationship for a particular material [21]. For instance, the surface roughness value up to Ra 0.3 μm was attained for the desired material through the meticulous selection of RUM variables [22]. Zheng et al. [23] developed an innovative composite diamond bit by integrating sintering and brazing for drilling holes in alumina (Al_2_O_3_), and silicon carbide (SiC). Low-frequency axial vibration technology was implemented with this bit, and the outcomes were compared against the traditional drilling process. It was realized that through this process axial force was significantly reduced and there were fewer plastic scratches on the hole wall. The automatic blanking ratio approaches 100% with this process as compared to 73.58% with conventional drilling. Nad et al. [24] examined the influence of vibration and tool shape on the edge chipping mechanism for rotary ultrasonic drilling (RUD). It was revealed that the highest value of corresponding von Mises stress increases as drill depth increases. The highest stress peaks were realized at the base of the external radius of the drilled hole. As reported in [25] the attrition mechanism of the diamond grains at the matrix labial surface can be classified into three phases: perfect crystal, trivial wear, and serious wear phases. The drill slipping phenomenon was observed during this investigation. It was noted that the grinding or wearing of the exposed diamond grains into the polished planar shape was the responsible factor for drill slipping. A research has been conducted on robotic rotary ultrasonic drilling (RRUD) to reduce the lateral vibration in the process. Using kinematic characteristics analysis, stability lobe diagram, and dynamic cutting force model the problem was analyzed. Then, a stability region was identified and validation experiments were conducted. It was reported that the stability region changes slightly with the variation of ultrasonic frequency. Moreover, the region is relatively bigger when the frequency is 20 KHz [26]. Kumar and Singh [27] implemented the TOPSIS (technique for order of preference by similarity to ideal solution) to optimize the RUD process parameters (feed rate, tool rotation speed, and ultrasonic power) for silica based glass BK7. Response variables under consideration were chipping width, taper, and material removal rate (MRR). The following optimized parameters were reported feed 0.60 mm/min, tool RPM 5000, and ultrasonic power 70%.

Jafarian et al. [28] conducted the experiments to optimize the surface quality and machining parameters while drilling holes in AISI H13 steel. Two regression models were developed, and then the nondominated sorting genetic algorithm (NSGA-II) was implemented to optimize the input variables. It was reported that an increase in cutting speed and liquid coolant intensity decreases the surface quality, while the higher depth of cut, tool diameter, and reed rate improve it. Elsen et al. [29] conducted a multi-objective optimization study for machining of alumina reinforced aluminum metal matrix composite utilizing response surface methodology (RSM). Chowdhury et al. [30] used the uncertainty analysis to predict the machining performance for a given input parameters settings of RUM. The workpiece material was Ti6Al4V. Multi-objective optimization of alumina bioceramic was performed for microRUM for milling microchannels. A multi-objective genetic algorithm (MOGA) was utilized for the optimization of input factors. Vibration frequency, amplitude, depth of cut, spindle speed, and feed rate were considered in the study. The output responses were surface roughness, side edge chipping, bed edge chipping, depth error, and width error [31]. Simultaneous optimization of MRR and surface roughness was performed for RUM parameters while machining quartz glass. Taguchi grey relational analysis was applied for multi-objective optimization. The results demonstrated that high MRR and low surface roughness for the quartz glass could be achieved with the tool rotational speed of 5000 rpm, feed-rate of 0.75 mm/min, and ultrasonic power of 55% [32].

A handful of research work has been conducted for the drilling process in ceramics, however, still, several issues on the hole’s poor dimensional accuracy, low MRR, fracture at hole exit, cylindricity, taperness, etc., persist. This study therefore aims to explore the impact of the main RUD process variables on the dimensional accuracy of the drilled holes. With an objective to enhance the machining of Al_2_O_3_ ceramics, drilling experiments were carried out. The RUD parameters, comprising vibration frequency, vibration amplitude, spindle speed, and feed rate have been investigated. A four-factor three-level central composite design (CCD) is employed to examine the relative significance of the mentioned RUD process parameters. The multi-objective genetic algorithm (MOGA-II) [33] has also been utilized to identify the optimal machining conditions for higher MRR, enhanced surface finish, and leading dimensional accuracy. It is essential to implement the MOGA-II approach for the RUD process because the output responses, most often, differ in their nature from each other. The verification tests have also been performed to validate the quality of drilled holes at the optimum process parameters. The main purpose of this research is the enhancement of the machining quality of Al_2_O_3_ ceramics using RUD.

## 2. Materials and Methods 

**Figure 2:**
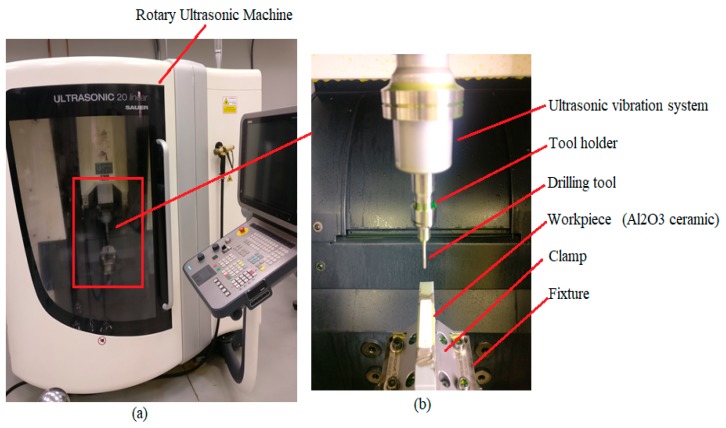

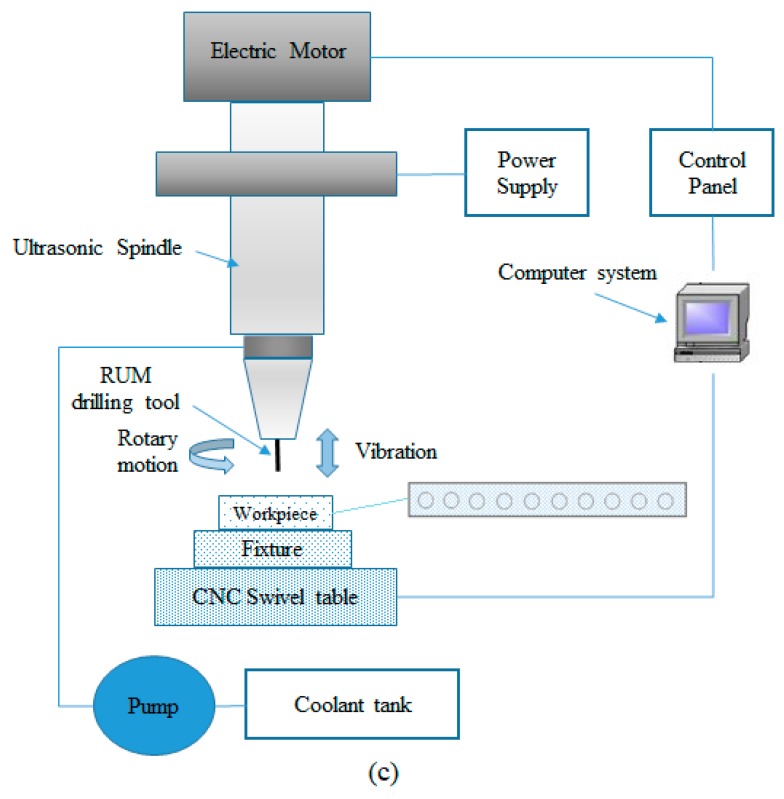
(**a**) DMG ultrasonic-20 linear machine, (**b**) experimental setup details, and (**c**) experimental setup schematics.

Ultrasonic 20 linear (DMG, Geretsried, Germany) was used to perform the experiments in this research work. It is a multi-axis machine with 5-axis capability (positioning accuracy of ±2.5 μm) that has both features (high-speed milling as well as drilling, and RUM). The major components of this machine are the HSK32 ultrasonic actuator system, an ultrasonic spindle attached to an ultrasonic transducer with a maximum rotation speed of 10,000 rpm, control system, power supply, coolant system, CN-rotary table, and the maximum amplitude of 80 µm. To generate ultrasonic frequency in the range of 18.5–48 kHz, and output peak power of 2 kWatts was used to convert a 50 Hz alternative current (AC) supply. Through an ultrasonic generator, the tool vibration amplitude (5–80 µm) was controlled in terms of the ultrasonic power percentages (50%–100%). Figure 2 shows the experimental setup with its schematic diagram.

### 2.1. Workpiece and Tool Description

A bioceramic (alumina Al2O3, 99.8%) that has application in the dental and medical field [34] was used as a workpiece material in this research work. The material was obtained from CeramTec (Plochingen, Germany) in the form of rectangular blocks. Then using the IsoMet® 1000 Precision Saw (Buehler, Illinois, US), samples of 50 mm × 10 mm × 3 mm were obtained. Three blocks of ceramic were realized. As obtained from the manufacturer, the critical characteristics of the specimen material are provided in Table 1. 

The RUD tool used in this study was made up of nickel and bonded with diamond particles. It is a hollow metal bonded diamond coated drills supplied by the Schott company (Mitterteich, Germany). The main specifications of the RUD tool are given in Table 2. The tool was changed throughout the experiments.

**Figure 3:**
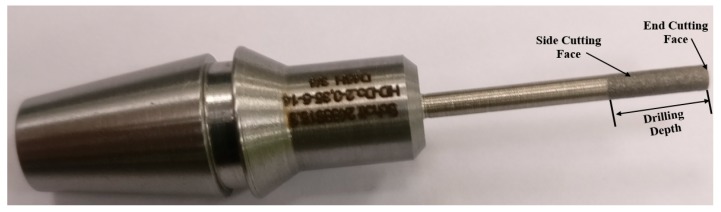
RUD tool.

Figure 3 shows the RUD tool used in this study.

In this setup, Grindex-10 (provided by Blaser swisslube, New York, NY, US) and deionized water were used as a coolant. 

**Figure 4:**
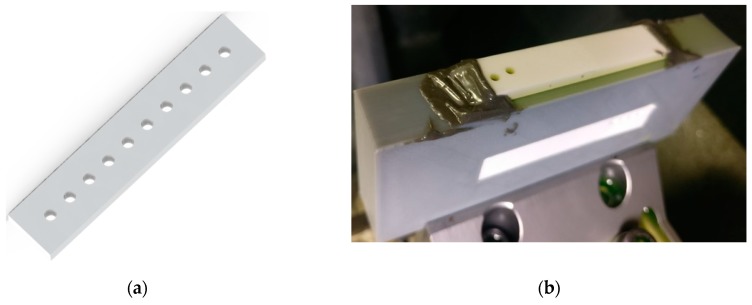
(**a**) Schematic of drilled holes and (**b**) actual machined holes in alumina ceramic.

Thorough holes of 2 mm were drilled in the workpiece. Figure 4 shows the schematic of the drilled holes and actual machined holes. A fixture was designed using 3D printing to hold the workpiece during RUD. 

### 2.2. Design of Experiments

In this research work, experiments were conducted depending on the CCD, so that the influence of input variables that are the speed, feed rate, amplitude, and frequency can be studied efficaciously on the output responses. Minitab 17, a statistical analysis software, was used for CCD formulation and analysis. Based on the constraints imposed by the experimental setup, preliminary runs, and the reported literature [17,31,35,36] on the drilling of brittle materials, the RUD parameters and levels were selected. Table 3 presents the input variables and their respective levels. The remaining parameters, including coolant type (grindex 10) and coolant pressure (4 bar) were constant across all the experiments. Based on CCD, 30 experiments were conducted at different input variables set. In a single work piece block, 10 holes were drilled.

Three output responses were considered to analyze the hole quality that are material removal rate (MRR), cylindricity, and taper angle. MRR is always considered as a vital response in any machining process since it is directly related to productivity. In this work, MRR is computed as the average volume of the specimen material dislodged to the corresponding machining time and is typically designated in cubic millimeters per minute (mm^3^/min). The generic volume formula considered for the MRR can be expressed as the volume of a conical frustum since there will be slight taperness. The mathematical expression for MRR calculation is as follows:

(1) $$\mathrm{MRR}=\frac{\left(\frac{\pi }{3}\right)*\left(R_{t}^{2}+R_{t}R_{b}+R_{b}^{2}\right)*h}{T}\;({\mathrm{mm}}^{3}/\min)$$

where *R_t_* represents the hole radius at the top, *R_b_* is the hole radius at the bottom, *h* is the workpiece thickness, and *T* is the machining time.

**Figure 5:**
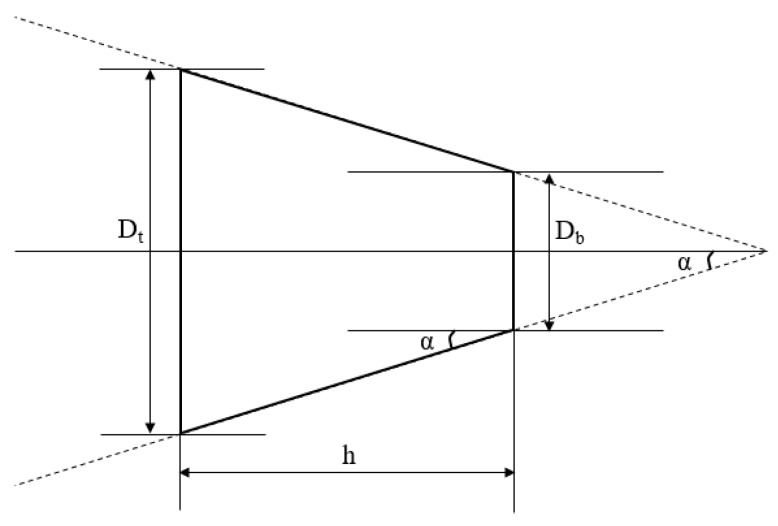
Taper angle calculation.

The cylindricity can be described as a three-dimensional tolerance that maintains the overall form of a cylindrical feature to assure that it is round and straight along its axis. Furthermore, the taperness can be defined as the difference between the entrance diameter and the exit diameter of the hole. The angle due to taperness is termed as the taper angle. Figure 5 shows the concept of taper angle calculation

(2) $$\alpha =\tan^{-1}(\frac{D_{t}-D_{b}}{2h})\;({}^{\circ})$$

where *D_t_* symbolizes the diameter of hole at the top, *D_b_* is the diameter of the hole at the bottom.

### 2.3. Response Measurement

**Figure 6:**
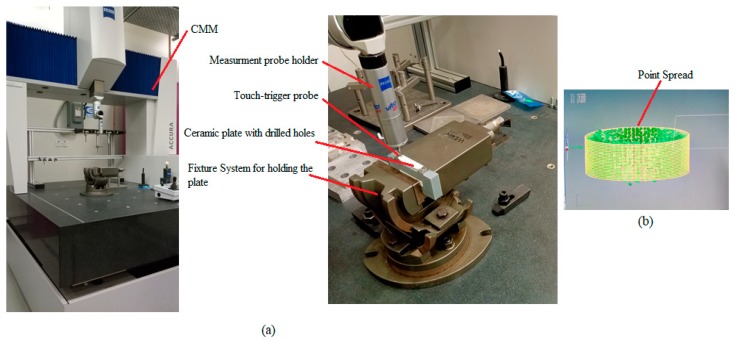
(**a**) Coordinate measuring machine (CMM) measurements system and (**b**) measurement point coverage.

The investigation of drilled holes for geometrical accuracy and taperness was achieved using a coordinate measuring machine (CMM). A bridge-type CMM (Zeiss, Oberkochen, Germany) integrated with a touch-trigger probe as depicted in Figure 6 was employed to assess the geometrical performance (cylindricity) of the drilled holes. Similarly, the diameters at the two extreme ends of the holes as well as their heights were also acquired for subsequent computation of taperness. All the holes were inspected thrice, and then their averages were calculated. As shown in Figure 6a, the parts were fixed on the CMM to carry out the measurements. The samples were mounted on a fixture that was uncomplicated and provided an immovable structure.

The inspection points allocated over 360° were employed to estimate the cylindricity as well as the taperness of different holes. A total of approximately 900 points were uniformly distributed along the entire face of the machined holes as depicted in Figure 6b. Although a minimum of two levels and six points (three points in each level) are acceptable to compute the cylindricity. However, in the current study, a vast number of points and levels that could mask the entire inspecting surface were considered. The touch probe used in this investigation had a tip radius of 0.5 mm and an overall length of 20 mm.

For qualitative analysis both an optical microscope (ASKANIA, Germany) and scanning electron microscope (SEM) were utilized. The drilled holes were analyzed qualitatively using the optical microscope first at 1.25× magnification. Both the entrance and exit of the holes were captured. For further investigation at optimized parameters images have been captured using a SEM (JEOL, Japan) to examine the drilled holes at 30× magnification. Since the workpiece material is ceramic, so to avoid the charging phenomenon in SEM, the specimens were coated with platinum.

## 3. Results and Analysis

The output responses were measured for each set of experiments. The design table with the measured output response is shown in Table 4.

**Figure 7:**
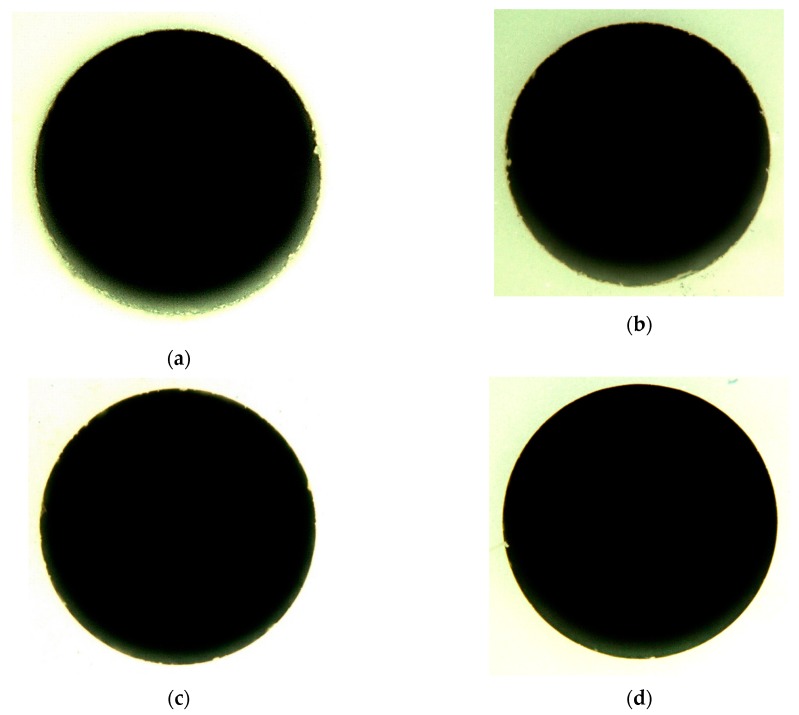
Image obtained through an optical microscope. (**a**) Top side of the hole (experiment #12). (**b**) Top side of the hole (experiment #14). (**c**) Bottom side of the hole (experiment #12). (**d**) Bottom side of the hole (experiment #14).

Figure 7 shows the images of some of the drilled holes obtained through an optical microscope at the 1.25× magnification level.

Holes 12 and 14 were chosen because they represent the median of input variables. From Figure 7, it could be deduced that the drilled hole quality was in good in terms of circularity as well as edge deformation. Both at the entrance side, and exit side the quality of holes was good. In addition, the dimensional accuracy was also verified by comparing the drilled (or machined) hole with the designed hole (2 mm diameter). The designed hole had zero cylindrical error and no taperness. The tolerances for cylindricity and taperness were 0.1 mm and 10° respectively. Therefore, it could be said that RUD was an efficient process to drill holes in a brittle and hard material such as alumina ceramic. 

### Multi-Objective Optimization

**Figure 8:**
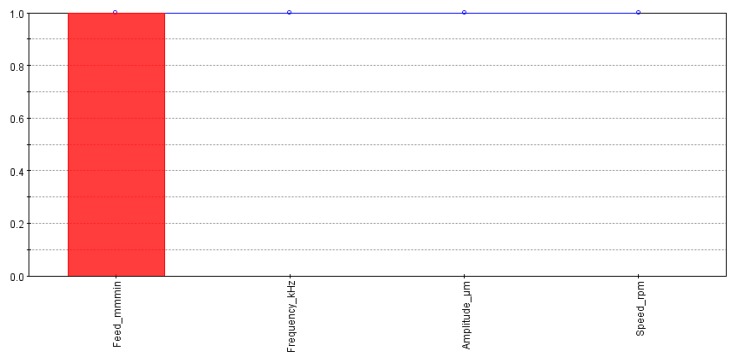
Effects of input parameters on the material removal rate (MRR).

To implement multi-objective optimization response surfaces are generated to predict the design points not available in the original design of experiment (DOE) plan. Radial basis functions (RBF) are selected to obtain response surfaces for the three output indicators. The results generated by RBF will be taken as input for the optimization solver. First, the influence of individual factors was analyzed on the output responses. The effects of different input variables on MRR are presented in Figure 8. It is quite clear that MRR is only dependent on the feed rate whereas other factors show no influence on the MRR.

**Figure 9:**
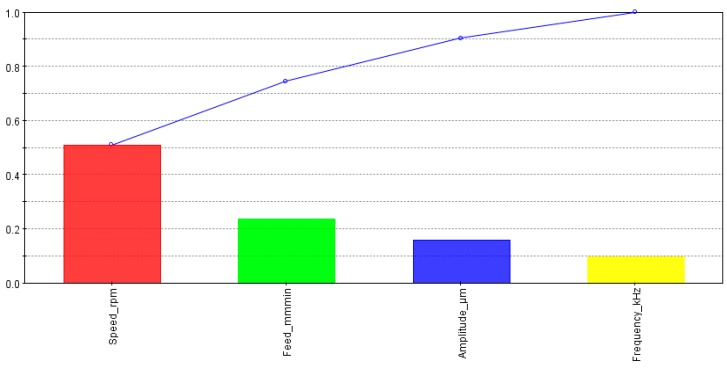
Effects of input parameters on cylindricity.

Figure 9 demonstrates the effects of input variables on the cylindricity of machined holes. The cylindricity of the holes was mostly controlled by the spindle speed and feed rate followed by amplitude and frequency. The effect of spindle speed was almost twice as the effect of the feed rate and approximately five times as compared to the effect of the frequency.

**Figure 10:**
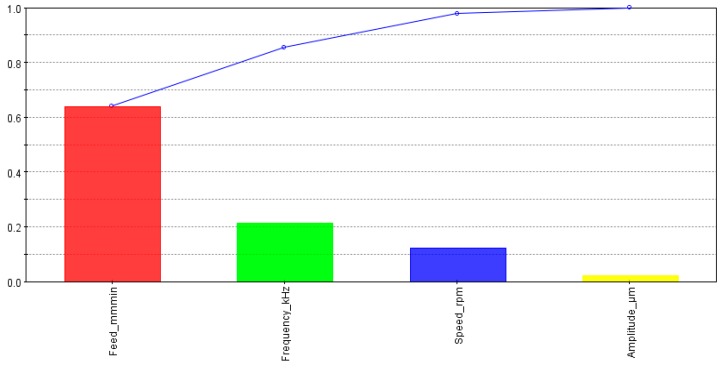
Effects of input parameters on taperness.

Figure 10 shows the response of input parameters on the taper angle. It could be visualized that the feed rate had the strongest influence on the taper angle followed by the frequency and speed. In contrast, the amplitude had a minimal effect on the taper angle.

**Figure 11:**
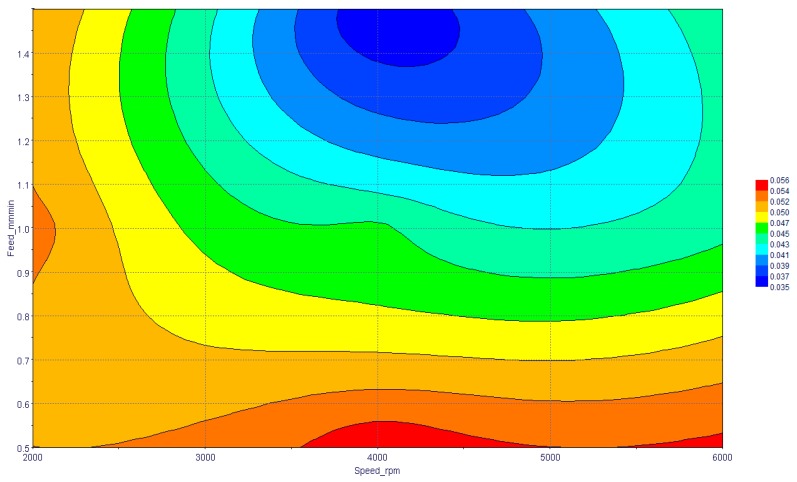
Combined effects of speed and feed rate on cylindricity.

In the next step, the combined effect of influencing factors was studied with the help of the response surface generated. Since MRR was affected by only a single factor, so it was not required to study the combined effect for it. The combined effect of the spindle speed and feed rate on the cylindricity of the machined holes is shown in Figure 11. It is depicted from the figure that optimum cylindricity was found to be at moderate spindle speed and high feed rate. At low feed rate, cylindricity error was high and it was not changing much by a variation in spindle speed. Similarly, at low spindle speed, the influence of feed rate was minimal but high variation can be seen at a moderate spindle speed.

**Figure 12:**
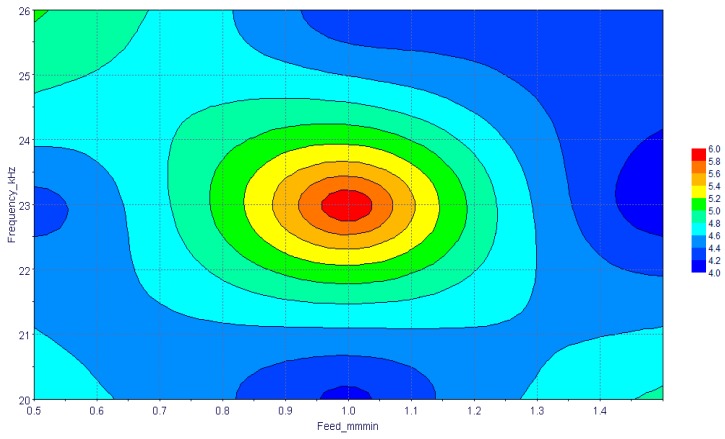
Combined effects of speed and feed rate on taperness.

Figure 12 presents the combined effect of the feed rate and frequency on the taper angle of the machined holes. Optimum taper angles were found at three locations as shown in Figure 12. Low taper angle regions could be visualized at higher feed rates with moderate to high frequencies. At the low feed rate, moderate frequency should be adopted and vice-versa for low taper angles. The effect of frequency was more pronounced at a moderate feed rate and the highest taper angle was found at a moderate frequency and feed rate.

Finally, a multi-objective optimization problem was utilized to maximize MRR and minimize cylindricity. Taper angles equal to and greater than 5 degrees are regarded as unfeasible. The optimization was performed using modeFRONTIER^®^ software, and MOGA-II, a multi-objective genetic algorithm was implemented as an optimization solver. It can be described as an effective algorithm that is based on a smart multisearch exclusiveness. The advanced exclusivity operator reserves a few supreme solutions without inducing early convergence to local optimal frontiers. MOGA-II needs very few user-provided variables for easiness and several other factors are inwardly resolved to provide the optimizer with stability and efficiency. The algorithm seeks all of the tests that are equivalent to the number of points in the DOE list (the initial population) multiplied by the number of generations. Readers can refer to the modeFRONTIER^®^ documentation for further technicalities and explanations on the MOGA-II [37]. A total of 3000 generations were sought in MOGA. The formulated optimization problem containing objective functions and constraints is depicted in Table 5. 

**Figure 13:**
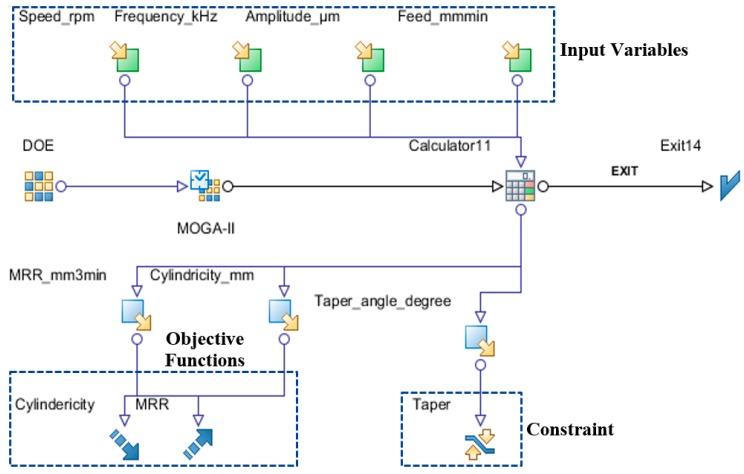
Optimization problem work flow.

The optimization workflow with input variables, objective functions, and constraint is shown in Figure 13.

**Figure 14:**
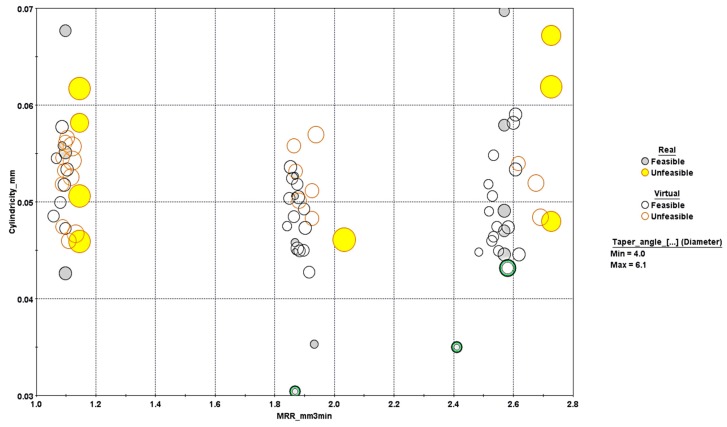
3D bubble chart with design points.

**Figure 15:**
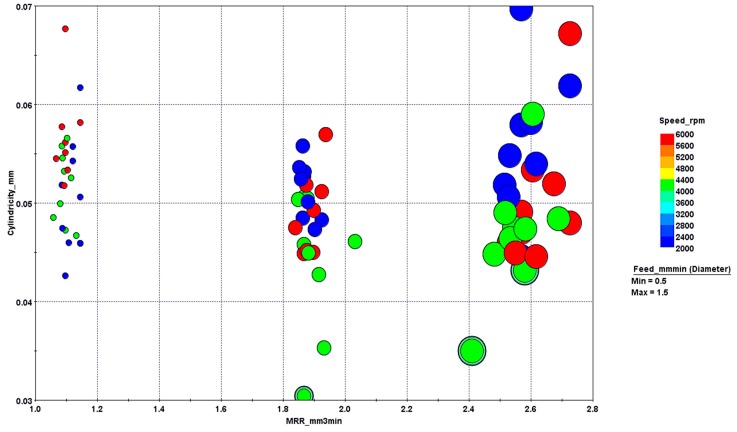
Effects of speed and feed rate in a 4D bubble chart.

The optimization problem was solved by the program, and the results were obtained in the form of charts and graphs. Figure 14 and Figure 15 show the design points acquired after optimization runs using 3D and 4D bubble charts. Virtual design points are those that are not contained in the DOE matrix. Unfeasible design points mean they violate the constraint, i.e., taper angles are equal to or greater than 5 degrees. Since the objective was to maximize material removal rate and minimize cylindricity, the design points at the lower right corner could be considered as optimal. The three points marked A, B, and C could be considered on Pareto-front showing the nondominated solutions. The distribution of design points showing the significance of two input parameters viz. spindle speed and frequency is shown in Figure 15. It was evident that low cylindricity was mostly associated with a moderate spindle speed and high feed values. The dependency of MRR on feed rate was pretty straight forward and the design points could be seen in three groups according to their feed values.

**Figure 16:**
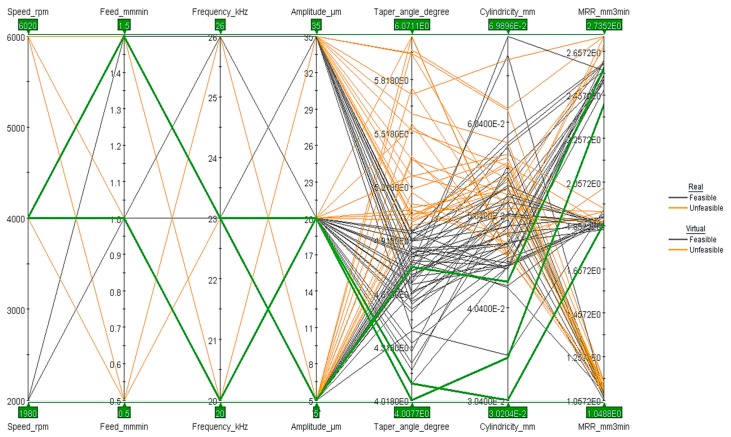
Parallel coordinate chart with all parameters.

The parallel coordinate chart as shown in Figure 16 represents another technique to examine all the design points. It could be realized that optimal design points were distinguished by a moderate spindle speed, moderate and high feed rates, and low to moderate frequency and amplitude. It represents the different design points with lines. The three green lines represent the optimal parameters settings. The details regarding the optimal design points are shown in Table 6.

**Figure 17:**
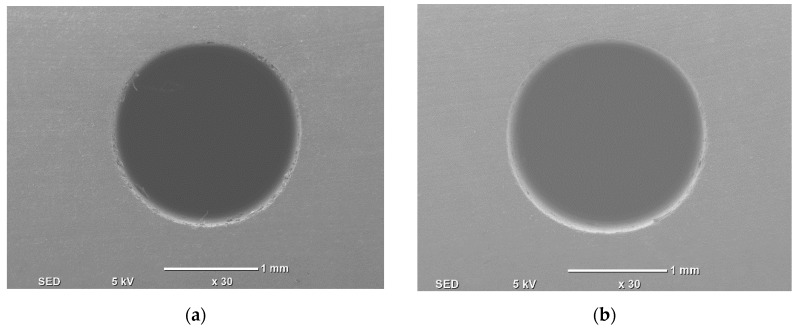

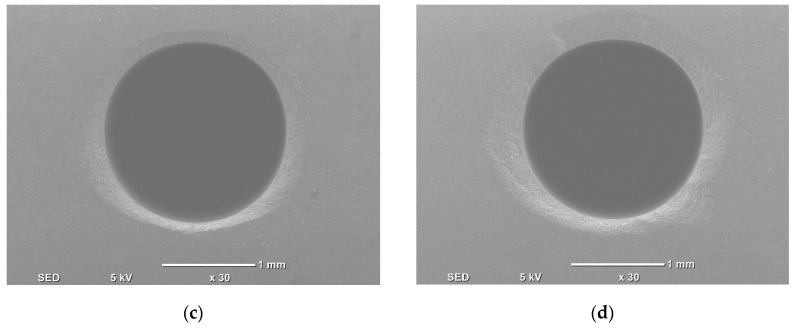
SEM image. (**a**) Top side of the hole (experiment #19). (**b**) Top side of the hole (experiment #21). (**c**) Bottom side of the hole (experiment #19). (**d**) Bottom side of the hole (experiment #21).

Figure 17 shows the SEM images of the top and bottom side of the holes at the 30× magnification level.

Holes #19 and #21 were selected since they were the results of the optimal parameters setting. Based on Figure 17 also, it could be said that hole dimensional quality at both sides was good. Moreover, dimensional accuracy was also maintained. There were no significant damages at the edges, i.e., there was no edge chipping and the smoothness of the surface was also achieved.

## 4. Discussion

In general there is a problem of the recast layer and dimensional inaccuracy with other nonconventional manufacturing processes such as a laser [12]. Therefore, in this research work, the RUD is investigated for the precise drilling of holes in Al_2_O_3_ ceramic. From the analysis it is evident that MRR was highly dependent on feed rates. This is due to the fact that the machining time decreased with increase in the feed rate (mm/min). Other researchers have also reported a similar type of observation. For example, Li et al. [18] conducted investigations for ceramic matrix composites and identified an improvement in MRR with a feed rate increase. Similarly, the outcome of the study carried out by Jiao et al. [38] and Wang and Chueh [39] is consistent with the findings of the current investigation. Kumar and Singh [36] have also suggested that the feed rate must be carefully chosen to achieve better machining efficiency. Furthermore, the MRR in RUM of quartz glass was found to be significantly affected by the feed rate [32]. It should be noted that in the present study the diameters of the holes at the entry and exit were positive (higher than the designed dimensions). This is because the effective diameter of the tool was always greater than the nominal diameter due to the variation in the abrasive sizes of the diamond bonded to cutting tools. This issue of overcut during RUM due to the variation of the diamond grit size was also encountered and explained by Abdo et al. [26]. In case of dimensional accuracy, it has been realized that the cylindricity of the holes is mainly dominated by the speed and feed rate of the spindles accompanied by the amplitude and frequency. In fact, the spindle speed influence was nearly twice as that of the feed rate effect, and about five times as opposed to that of the frequency effect. The optimum cylindricity was noticed with a moderate spindle speed and high feed rate. At a low feed rate, cylindricity error was high. This implies that any combination of the RUM parameters that reduces the cutting forces such as higher amplitudes, low feed rate, etc., provide lower dimensional error. Moreover, the cylindricity error could be attributed to the fact that the buildup of debris in the machining gap was high at a low feed rate and low spindle speed, due to which the movement of debris (or chips) in the machining zone was impeded. As a consequence, thick layers of debris were formed, thereby decreasing the machining rate significantly, increasing the tool wear and deteriorating the dimensional accuracy. It can also be seen that the feed rate had the greatest impact on the taper angle followed by frequency and speed. The amplitude had a marginal effect on the angle of the taper. The taperness was caused by a larger diameter at the entrance and a relatively small diameter at the exit sides. It might be because of chipping and higher damage at the entrance side, while a very little damage at the exit side. It can be inferred that higher cutting forces and highly unpredictable initial process phase may have damaged the surface at the start of the drilling. However, with the advancement of the tool in the workpiece surface the cutting forces and the process is stabilized to minimize the effect of chipping. In addition, the trigger for taper formation tends to be inadequate debris movement and specimen wall cracking at a higher depth. A further explanation for cylindricity error and taperness is edge chipping due to excessive centrifugal force induced by tool rotation outward from the tool axis. Certainly, the particles scraped away the wall and the perimeter, while withdrawing through the hole, thereby causing dimensional inaccuracy.

The competent methodology embraced in this work can be employed to achieve optimal parameter combination for drilling holes in any ceramic material. This research can potentially be applied in many areas, including the dental industry, the automotive industry, the aerospace industry, etc. However, the limitation of this study was the absence of the investigation of the tool wear affect. Due to the high cost of the experimentation, the tool wear was not considered in this work. Nevertheless, tool wear is an unavoidable event in the RUD process. Therefore, it becomes mandatory to examine the tool wear because it noticeably influences the machining performance and its cost. Henceforth, this work will be extended in the future to incorporate the tool wear study. Another drawback of this work was RUD’s low efficient machining for ceramics, which will need more detailed investigations in the years ahead. Only one type of ceramic workpiece material, tool material as well as fixed dimensions of the holes and specimen were studied. Consequently, in order to extend this work, the RUD process will be explored for the machining of different types of ceramic materials using distinct tooling materials for varying hole dimensions. 

## 5. Conclusions

In this research work, an experimental study was executed to evaluate the effect of major RUD input variables on MRR, and hole dimensional accuracy while drilling on advanced ceramic alumina (Al_2_O_3_). Based on this research work the following conclusions could be deduced:

- RUD is an efficient process to drill accurate and precise holes in difficult to cut brittle materials such as ceramics.
- Results showed that MRR was only dependent on the feed rate, whereas cylindricity of the holes was mostly controlled by the spindle speed and feed rate. Moreover, the feed rate exhibited the strongest influence on the taper angle followed by the frequency and speed.
- MOGA-II is a competent method to deal with the multi-objective problem and obtains the optimal parameters. The optimal parametric combination of RUD to achieve good quality holes in alumina with high MRR and accuracy were as follows: speed = 4000 rpm, feed rate = 1.5 (mm/min), amplitude = 20 (µm), and frequency = 23 (kHz).

## Figures and Tables

**Table 1:** Workpiece material characteristics (as provided by the manufacturer [34]).

Material Property	Value (units)
Flexural strength (20 °C)	1100 (MPa)
Compressive strength	5500 (MPa)
Tensile strength	650 (MPa)
Poisson’s Ratio	0.23
Bulk Density	3.96 (g/cm^3^)
Young’s modulus	406 (GPa)
Vickers hardness (HV 0.5)	2000
Thermal conductivity (20 °C)	30 (W/mK)
Melting point	2270 (°C)

**Table 2:** Rotary ultrasonic drilling (RUD) tool specifications.

Tool Type	Outer Dia. (mm)	Wall Thick. (mm)	Drilling Depth (mm)	Grain Size	Binding
Hollow drill	2	0.35	7	D46	GVD

**Table 3:** Input variable and their levels.

Input Variables	Levels		
	1	2	3
Speed (rpm)	2000	4000	6000
Feed rate (mm/min)	0.5	1	1.5
Vibration Amplitude % (µm)	5	20	35
Vibration Frequency (kHz)	20	23	26

**Table 4:** Design table with output responses.

	Input Parameters				Output Response		
Exp. No.	Speed (rpm)	Feed (mm/min)	Amplitude (µm)	Frequency (kHz)	MRR (mm^3^/min)	Cylindricity (mm)	Taper Angle (°)
1	2000	0.5	5	26	1.14	0.0459	6.0599
2	2000	0.5	35	26	1.14	0.0617	5.9660
3	2000	0.5	35	20	1.14	0.0506	5.9639
4	2000	0.5	5	20	1.10	0.0426	4.8421
5	2000	1	20	23	1.87	0.0527	4.1882
6	2000	1.5	5	20	2.73	0.0619	5.9835
7	2000	1.5	5	26	2.57	0.0579	4.7539
8	2000	1.5	35	26	2.57	0.0446	4.8677
9	2000	1.5	35	20	2.57	0.0697	4.6744
10	4000	0.5	20	23	1.09	0.0558	4.2910
11	4000	1	20	23	2.03	0.0461	6.0609
12	4000	1	20	23	2.03	0.0433	5.7141
13	4000	1	20	23	1.94	0.0648	4.7291
14	4000	1	20	23	1.93	0.0747	4.9370
15	4000	1	5	23	1.93	0.0353	4.4078
16	4000	1	35	23	1.87	0.0458	4.4141
17	4000	1	20	23	1.87	0.0549	4.3294
18	4000	1	20	23	1.87	0.0511	4.3976
19	4000	1	20	20	1.87	0.0304	4.1106
20	4000	1	20	26	1.87	0.0506	4.2290
21	4000	1.5	20	23	2.41	0.035	4.0180
22	6000	0.5	5	26	1.14	0.0459	5.5726
23	6000	0.5	35	20	1.14	0.0582	5.6284
24	6000	0.5	35	26	1.10	0.0551	4.9216
25	6000	0.5	5	20	1.10	0.0677	4.7890
26	6000	1	20	23	1.87	0.0449	4.1106
27	6000	1.5	5	20	2.73	0.048	5.7730
28	6000	1.5	35	20	2.73	0.0672	5.7348
29	6000	1.5	5	26	2.57	0.047	4.7488
30	6000	1.5	35	26	2.57	0.0491	4.9113

**Table 5:** Objective functions and constraints of the formulated optimization problem.

Objective Functions	(1) Maximize MRR
	(2) Minimize Cylindricity
Constraint	Taperness ≤ 5°

**Table 6:** Optimal solution points.

S.No.	Speed (rpm)	Feed (mm/min)	Amplitude (µm)	Frequency (kHz)	MRR (mm^3^/min)	Cylindricity (mm)	Taper Angle (°)
A	4000	1	20	20	1.867	0.0304	4.1106
B	4000	1.5	20	23	2.4098	0.0350	4.0180
C	4000	1.5	5	23	2.5794	0.0432	4.7656
